# A Small Glomus Tympanicum Tumor Resected by Minimally Invasive Transcanal Endoscopic Approach

**DOI:** 10.1155/2019/5780161

**Published:** 2019-07-09

**Authors:** Masafumi Ohki, Shigeru Kikuchi

**Affiliations:** Department of Otolaryngology, Saitama Medical Center, Saitama Medical University, 1981 Kamoda, Kawagoe-shi, Saitama 350-8550, Japan

## Abstract

We present a case of the transcanal endoscopic resection of a glomus tympanicum tumor. A 51-year-old woman presented with pulsatile tinnitus of the right ear persisting for 6 months. A reddish mass was observed through her tympanic membrane. A computed tomography scan revealed a small mass in the mesotympanum. She was diagnosed with a right-sided glomus tympanicum tumor. The glomus tympanicum tumor was classified as type 1 using the Glasscock–Jackson classification, class A using the Fisch classification, and class A1 using the modified Fisch and Mattox classification. The tumor was transcanally and completely resected by endoscopy without any complication. Before and after the surgery, pure-tone audiometry showed a normal hearing level. Preoperative right-sided pulsatile tinnitus resolved after the surgery. Transcanal endoscopic ear surgery is a favorable surgical method for small localized glomus tympanicum tumors.

## 1. Introduction

Glomus tumors are rare neuroendocrine tumors arising paraganglionic cells throughout the body. Glomus tumors within the temporal bone are classified as either a glomus tympanicum tumor or a glomus jugulare tumor [1].

Complete surgical resection is a common treatment. There are various approaches and strategies: transcanal approach, postauricular approach, canal wall-up mastoidectomy with posterior tympanotomy, canal wall-up mastoidectomy with posterior tympanotomy and subfacial recess tympanotomy, and subtotal petrosectomy with middle ear obliteration [2, 3]. These surgeries may be adopted depending on the type, size, and site of the tumor; patient's conditions; and surgeon's skills. These surgeries have been commonly performed under microscopy. Recently, transcanal endoscopic ear surgery has been established as the treatment modality for chronic otitis media [4, 5]. Endoscopy has the major advantage of having a wider angle of view than microscopy. Herein, we report the minimally invasive transcanal endoscopic resection of a glomus tympanicum tumor.

## 2. Case Presentation

**Figure 1:**
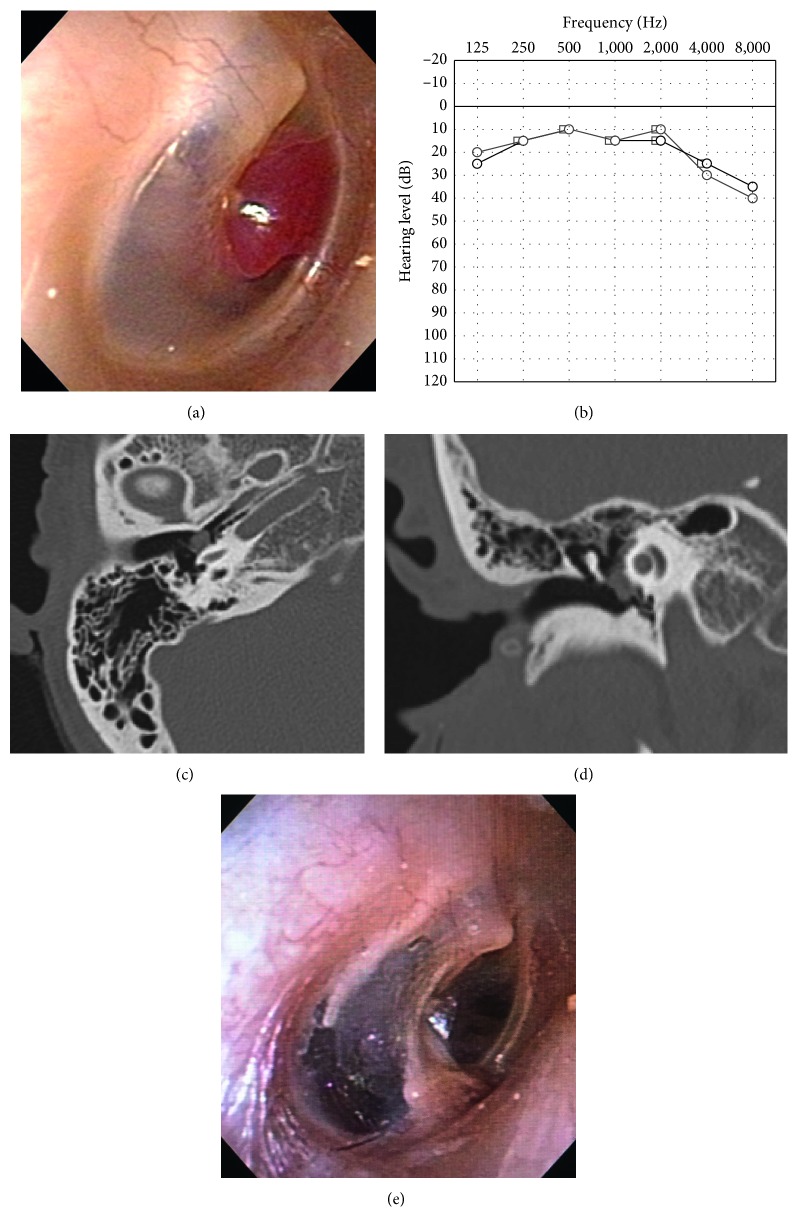
(a) A red mass was observed under the anterior quadrant of the tympanic membrane. (b) Pure-tone audiometry. The black and gray lines show the preoperative and postoperative hearing levels, respectively, in the right ear. After the surgery, the hearing level was not reduced and a normal hearing level was preserved. (c, d) A high-resolution computed tomography scan ((c) axial view and (d) coronal view) shows a mass on the promontory. (e) Postoperative tympanic membrane.

**Figure 2:**
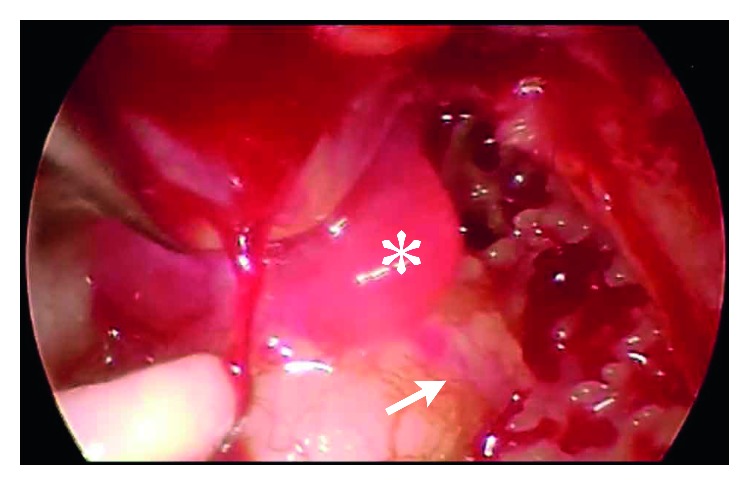
Findings seen during transcanal endoscopic ear surgery. The reddish tumor (asterisk) was located on the promontory. The tumor feeder was identified as the inferior tympanic artery (arrow).

**Figure 3:**
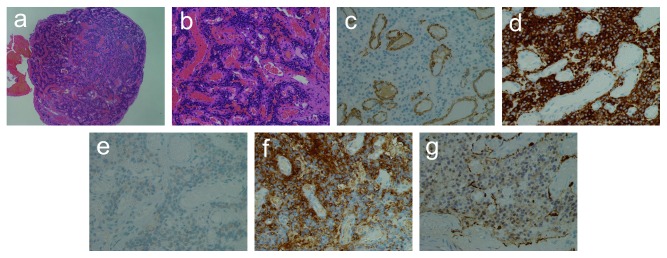
Histopathology. Hematoxylin–eosin staining: (a) reticular vascular-rich round tumor (×40) and (b) round tumor cells were aggregated showing ribbon-like (arrow) and island-like (arrow head) formations in vascular spaces (vascular spaces were lined by endothelial cells (×200)). Immunohistochemical staining (×400): (c) *α*-smooth muscle actin (*α*-SMA) was negative, (d) synaptophysin was positive, (e) S-100 protein was positive on sustentacular cells, (f) cytokeratin AE1/AE3 was negative, and (g) neuron-specific enolase (NSE) was positive.

A 51-year-old woman presented with pulsatile tinnitus of the right ear persisting for 6 months. She had not experienced vertigo. A reddish mass was observed through the anterior quadrant of her right tympanic membrane (Figure 1(a)). The surface of the tympanic membrane was intact. Her pure-tone audiometry showed a normal hearing level on the right side (Figure 1(b)). The pharynx was intact, as observed using a fiberscope. A computed tomography scan revealed a small mass in the mesotympanum (Figures 1(c) and 1(d)). Aberrant arteries or high jugulars were not observed. She was diagnosed with a right-sided glomus tympanicum tumor. The tumor was localized on the promontory; therefore, it was classified as type 1 using the Glasscock–Jackson classification [2], class A using the Fisch classification [5], and class A1 using the modified Fisch and Mattox classification [3]. For this small localized glomus tympanicum tumor, transcanal endoscopic resection was performed, with microscopy surgical equipment as standby to be able to quickly shift to microscopic surgery. A bipolar cautery was also prepared. The skin of the external ear canal was incised, and the tympanomeatal flap was elevated, concentrating on not damaging the surface of the tumor to prevent bleeding (Figure 2). The tumor had spread over the promontory, along the tympanic nerve, and under the tympanic membrane. The tumor contacted the tympanic membrane but did not adhere to it. The vascular supply to the tumor was from the inferior tympanic artery (Figure 2). The superior, anterior, posterior, and inferior extents of the tumor were just below the horizontal portion of the facial nerve, Eustachian tube, posterior edge of the promontory, and inferior half of the promontory, respectively. The tumor was gently removed from the promontory. Next, the tympanic nerve and inferior tympanic artery were cut. Hemostasis was accomplished by securely packing with cotton and 0.02% epinephrine (Bosmin; Daiichi Sankyo Company Limited, Tokyo, Japan). Complete resection of the tumor was endoscopically confirmed. The tympanomeatal flap was returned to its original position. The total surgical time was 66 min, and the total bleeding volume was less than 10 ml. No cranial neuropathy occurred. Pathological findings (Figure 3) confirmed a reticular vascular-rich tumor (approximately 5 × 5 mm) comprising round tumor cells aggregating as ribbon-like and island-like formations in vascular spaces. The round tumor cells immunohistochemically stained negative for *α*-smooth muscle actin, positive for synaptophysin, negative for cytokeratin AE1/AE3, and positive for nonspecific esterase. Sustentacular cells were stained positive for S-100 protein. The tumor pathology was consistent with a paraganglioma. Postoperative pure-tone audiometry demonstrated a normal hearing level (Figure 1(d)). The patient's right-sided pulsatile tinnitus completely vanished postoperatively. No recurrence was observed 3 years after the surgery (Figure 1(e)).

## 3. Discussion

Gary Jackson [6] and Sanna et al. [3] reported that a localized glomus tympanicum tumor (type 1 using the Glasscock–Jackson classification [2] and class A1 using the modified Fisch and Mattox classification [3]) can be transcanally resected. Microscopic ear surgeries have been commonly performed for a glomus tympanicum tumor. Recent advances in endoscopy enable endoscopic ear surgery. Marchioni et al. [7] first reported transcanal endoscopic surgery for middle ear neoplasms including three cases of glomus tumor in 2013. Isaacson and Nogueira [8] and Killeen et al. [9] also described endoscopic surgery for glomus tumors. All groups performed transcanal endoscopic surgery for small glomus tympanicum tumors. The stages of a glomus tympanicum tumor removed by Marchioni et al. [7] were a modified Fisch and Mattox classification stage A2 tumor in two cases and a stage A1 tumor in one; Isaacson and Nogueira [8] removed a stage A1–B1 tumor. Killeen et al. [9] resected a stage A1-2 tumor. Marchioni et al. [7] performed degloving of the external auditory canal following enlargement of the bone annulus in a stage A2 tumor and a tympanomeatal flap technique in a stage A1 tumor. He mentioned that the degloving technique can improve the visualization of the posterior tympanic, protympanic, hypotympanic, or attic spaces for larger tumors involving these hidden areas and create a wider surgical space to allow a second operator to participate during the surgery; the tympanomeatal flap technique is for smaller lesions.

Transcanal endoscopic ear surgeries have advantages and disadvantages. Advantages are having a wide surgical view for approaching hidden areas such as the posterior tympanic cavity, inferior tympanic cavity, anterior tympanic cavity, and attic; looking behind corners; high magnification; managing the lesion also medially to the ossicular chain; the possibility to maintain, in some cases, the ossicular chain, detaching the tumor from the ossicles; and checking recesses to identify eventual residual disease [3, 7–9]. Major disadvantages are the need for one-handed maneuvering and the ease of fogging. The management of the flap and ossicular chain is important. To most visualize the tumor and ossicular chain, the flap should be detached from the malleus. Currently, transcanal endoscopic ear surgeries are suitable for small tumors, i.e., modified Fisch and Mattox classification stage A1 tumors, similar to the tumor in our case. In our case, a limited case of a tiny tumor, the tumor extension could be visualized and the tumor was confirmed to be free from the ossicular chain by the tympanomeatal flap technique under the endoscope; therefore, the flap was not detached from the malleus. If the resection underwent under a microscope, it is considered that the extension of the tumor to the anterior and superior direction could not confirmed by the tympanomeatal flap technique without the degloving technique. If the tumor is suspicious to attach with the ossicular chain, or the tumor extension cannot be easily visualized by the tympanomeatal flap technique, the tympanomeatal flap should be detached from the malleus to visualize areas behind the ossicles. The removal of stage A2–B1 tumors is still challenging as it is difficult to control hemostasis during one-handed manipulation. Therefore, the use of a coagulator and drilling of the overhanging bone for visualization are required. Marchioni et al. [7] and Isaacson and Nogueira [8] used bipolar microforceps to reduce the dimensions of the masses and avoid possible bleeding. The advantages of coagulation are the bleeding prevention and hemostasis to keep the surgical field clean and the reduction of the tumor volume. The use of coagulation for resection of a glomus tumor is important, the localization of the pedicle of the tumor with the coagulation of the vascular supply is mandatory, and the volume of the tumor can be reduced to manage the extension of the mass [3, 7–9]. Especially during endoscopic surgery is mandatory to keep the endoscope clean and to proceed with removal. In our case, a bipolar cautery should have been used to shrink the tumor and coagulate the feeding vessels because cotton soaked in epinephrine is less effective than a bipolar cautery. The other devises for coagulation, e.g., a CO_2_, diode, or KTP laser, were also reported to be useful [7, 9].

Isaacson and Nogueira [8] succeeded in exclusively performing endoscopic surgery in 10 of 13 cases but needed microscopy in three cases. Killeen et al. [9] reported that 79% cases (11 of 14) had complete resection via an exclusive endoscopic approach and the size of tumor was 3–15 mm. The other 3 cases needed transition to microscopic surgery. Standby microscopy is necessary and important for glomus tumor surgery. Larger glomus tympanicum tumors require a two-handed operation to control bleeding; therefore, microscopic surgery is commonly used. In case of microscopic surgery, an endoscope can assist in observing hidden areas.

Rich vascularity is a common feature of glomus tumors; therefore, angiography and/or embolization of the feeder artery are often preoperatively performed to identify the feeder artery, reduce the risk of bleeding, and reduce the tumor size [10]. Embolization is expected to reduce the total bleeding volume during surgery. However, embolization also carries the risk of infarction [10]. Cranial neural deficits after embolization are possible because of various anastomoses present between the external and internal carotid arteries [10]. Persky et al. [10] stated that the removal of a glomus tympanicum tumor does not always require preoperative embolization if surgery is performed by an experienced otologic surgeon. However, the decision depends on the location and extent of the tumor.

In conclusion, transcanal endoscopic ear surgery is a favorable surgical method for small localized glomus tympanicum tumors.
